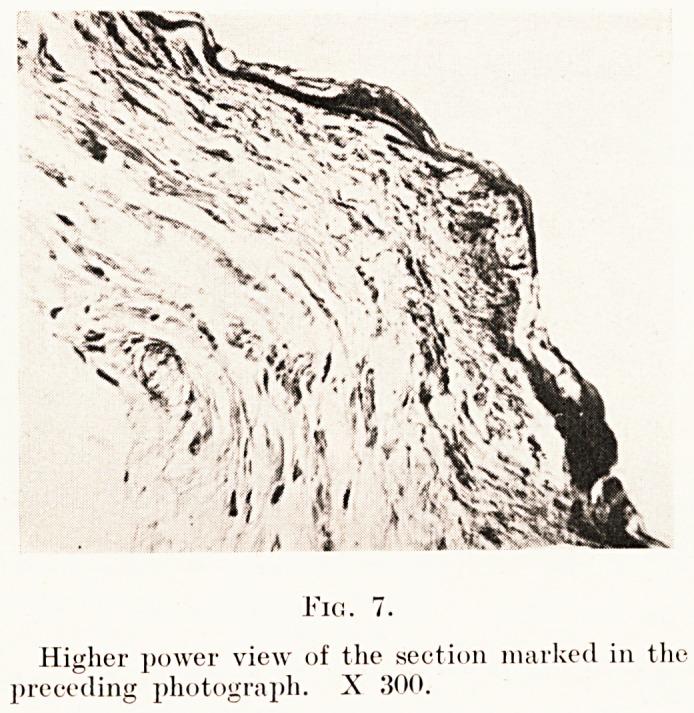# Senile Arterial Changes in a Child Aged Seven Weeks
*From the University Centre of Cardiac Research, General Hospital, Bristol.


**Published:** 1929

**Authors:** F. W. Terrell Hughes, C. Bruce Perry


### SENILE ARTERIAL CHANGES IN A CHILD AGED SEVEN WEEKS.*

BY

F. W. Terrell Hughes, M.B., Ch.B.,

AND

C. Bruce Perry, M.D., M.R.C.P.†

---

The following case of sudden death in a child is worthy of record in view of the curious pathological findings.

*History.*—The child, a girl aged seven weeks, was left for a few minutes in her perambulator in the care of her brother, while the mother prepared her feed for her. The brother called out that baby was "making a funny noise," and when the mother reached her she found her blue and obviously distressed. She picked her up and, thinking she "had the wind," turned her on to her face; the child, however, died almost immediately.

*Post-mortem Examination.*—A post-mortem examination was made by one of us (F. W. T. H.) at the coroner's request on the following day. The body was slightly cyanosed, but otherwise there was nothing abnormal externally. The only organ which presented an abnormal appearance was the heart. This, together with the thymus and lungs, was preserved for further examination.

The *heart* was normal in size, but attracted attention by the marked thickening and tortuosity of the coronary arteries, which stood out from the rest of the heart like cords. On opening the heart the only abnormality to be found was a valvular patency of the foramen ovale. All four valves were normal in appearance, as also were the aorta and pulmonary artery.

On microscopical examination the coronary arteries (see Figs. 2, 3) present an extraordinary picture. In all the main

* From the University Centre of Cardiac Research, General Hospital, Bristol.

† Working under a Colston Research Scholarship.

branches the lumen is more or less obliterated by extreme intimal thickening. This intimal thickening is composed of a loosely-arranged fibrillar connective tissue. There is no evidence of vascularization. The media is almost completely calcified and only very broken fragments of muscle fibres are left. The adventitia shows some increase in its fibrous tissue. This change is confined to the larger branches of both coronary arteries, the smaller arteries being normal. Scattered throughout the myocardium are areas of early fibrosis replacing the muscle fibres. The thoracic aorta (Fig. 4) shows patches of intimal proliferation and thickening, but sections of the ascending aorta and the arch are normal. The pulmonary artery presents small areas of calcification in the media. These are distributed throughout the thickness of the media, but are perhaps more frequent in the inner third and just below the intima. The vessels of the adventitia and the vasa vasorum of the outer third of the media are congested. The intima is normal.

The *lungs* were normal in appearance and well expanded and ærated. On section some of the smaller branches of the pulmonary artery show small patches of subintimal calcification. The deposit of calcium almost appears to have occurred in the elastic laminæ, and in sections stained with orcein looks like normal elastic tissue, but with hæmatoxylin stains the deep blue black colour of calcium. (See Figs. 5, 6, 7.)

The *thymus* was normal in size and appearance. On section it shows no abnormality and the arteries are normal.

*Family History.*—There is no history of heart disease in the family. The father is aged 32 and healthy. Five years ago he developed a pneumococcal septicæmia following a quinsy, from which he made a good recovery. The mother is aged 31 and healthy—the Wassermann reaction is negative.

This was the third child. The two others are a boy aged 7 and a girl aged 5, both quite healthy. There have been no miscarriages. The pregnancy was normal, except that the mother had an attack of "influenza" at the sixth month. The labour was conducted by a midwife, and was normal. The child was breast fed till six weeks old, when, the breast milk failing, she was given a mixture of cow's milk and water. She had never been seen by a doctor.

*Discussion.*—There is no evidence in any of the tissues examined of an inflammatory origin of the changes, which appear to be the result of some toxic

### PLATE XXIV.

FIG. 1.

General view of the heart, showing the thickened and tortuous coronary arteries.

FIG. 2.

Photomicrograph showing two branches of the coronary artery in cross section. X 25.

FIG. 3

Photomicrograph showing a higher power view of a coronary artery. X 25.

A The narrowed lumen.
B The thickened intima.
C The calcified media.
D The adventitial fibrosis.

FIG. 4.

Photomicrograph of a section of the descending aorta stained with orcein, showing the intimal thickening (X). X 75.

### PLATE XXV.

Fig. 5.

Photomicrograph of main branch of pulmonary artery, showing area of medial calcification. X 100.

Fig. 6.

Photomicrograph of a small artery in the lung showing sub-intimal calcification (X). X 75.

Fig. 7.

Higher power view of the section marked in the preceding photograph. X 300.

influence. It is unfortunate that the other visceral and peripheral arteries were not available for examination; but the change, though varying in intensity in the different organs and affecting the coronary circulation most severely, would appear to be generalized. From the degree of calcification which has taken place in the coronary arteries, one is forced to conclude that the process commenced during intra-uterine life. What was the cause it is obviously impossible to say. Were the young arteries exposed to, and damaged by, some circulating toxin, to which the more mature maternal arteries were immune, when the mother was ill with "influenza"? It is interesting to note that, except in the foci of maximum intensity, the medial and intimal changes do not coincide. The thoracic aorta, which shows intimal thickening, presents a normal media, while the pulmonary artery with scattered areas of medial calcification shows no intimal change. The two changes, which are, presumably, due to the same ætiological factor, thus develop quite independently, and cannot be considered in any way compensatory to each other. The only cases at all similar to this that we have been able to find in the literature are those described by Branson and McMichael respectively. Branson's case[1] was that of a child in whom all the smaller arteries and arterioles were the seat of a "proliferating arteritis." The possibility of syphilis was not definitely excluded, although Branson did not consider it likely. McMichael[2] describes the case of a child aged 18 months who died fairly suddenly after a prolonged indefinite febrile illness. The smaller arteries (the size of the coronaries and their main branches) showed "great connective tissue proliferation internal to the internal elastic lamina." The media, adventitia and the vasa vasorum were normal, and the smaller arterioles were unaffected. There was no evidence of syphilis or tuberculosis. In our case, too, the family history and the mother's Wassermann reaction rule out syphilis fairly definitely as a cause, but it differs from McMichael's case in the involvement of the media.

In conclusion, our thanks are due to Dr. A. L. Taylor for performing a Wassermann reaction on the mother's blood, and for much other help and advice.

REFERENCES.

[1] Branson, *Tr. Path. Soc.*, London, 1905, lv., 212.

[2] McMichael, *Arch. Dis. Child*, 1929, iv., 22, 165.

## Figures and Tables

**Fig. 1. f1:**
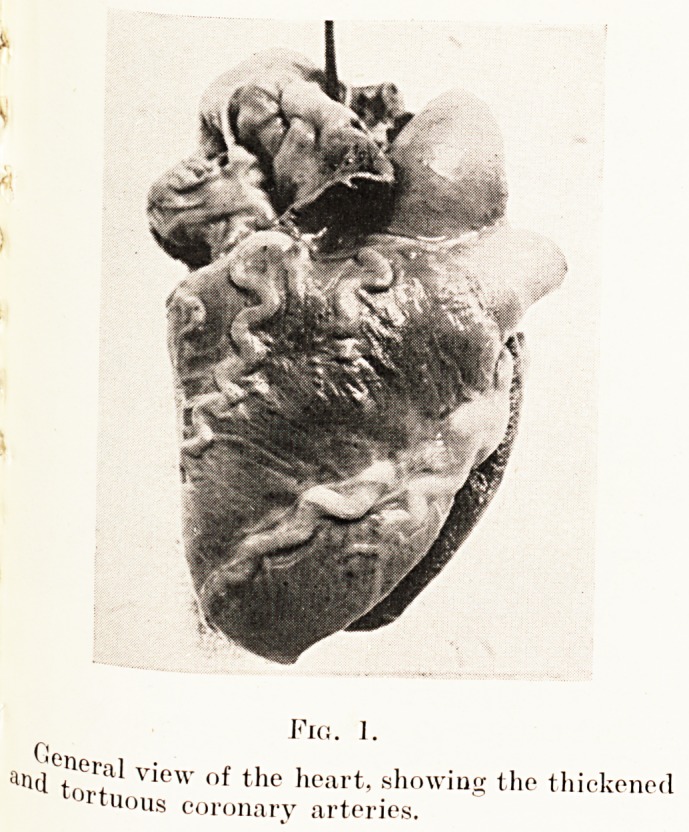


**Fig. 2. f2:**
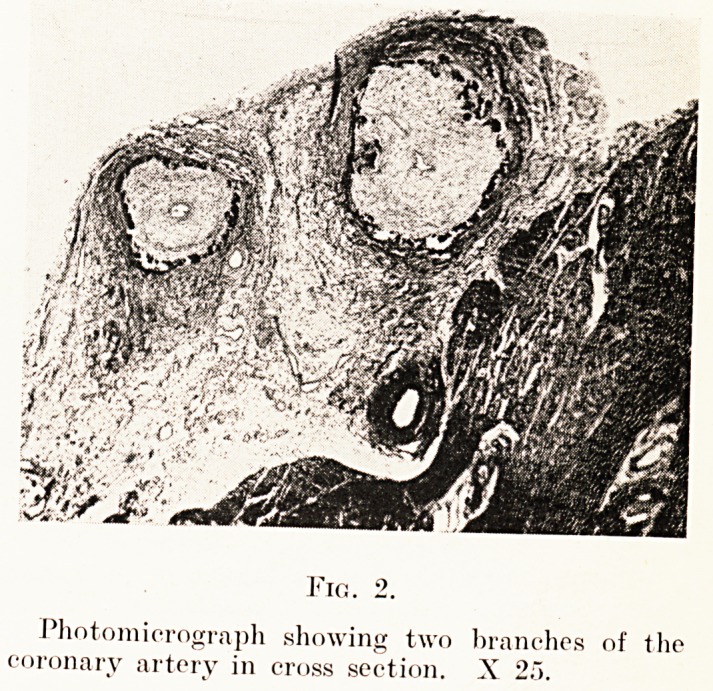


**Fig. 3. f3:**
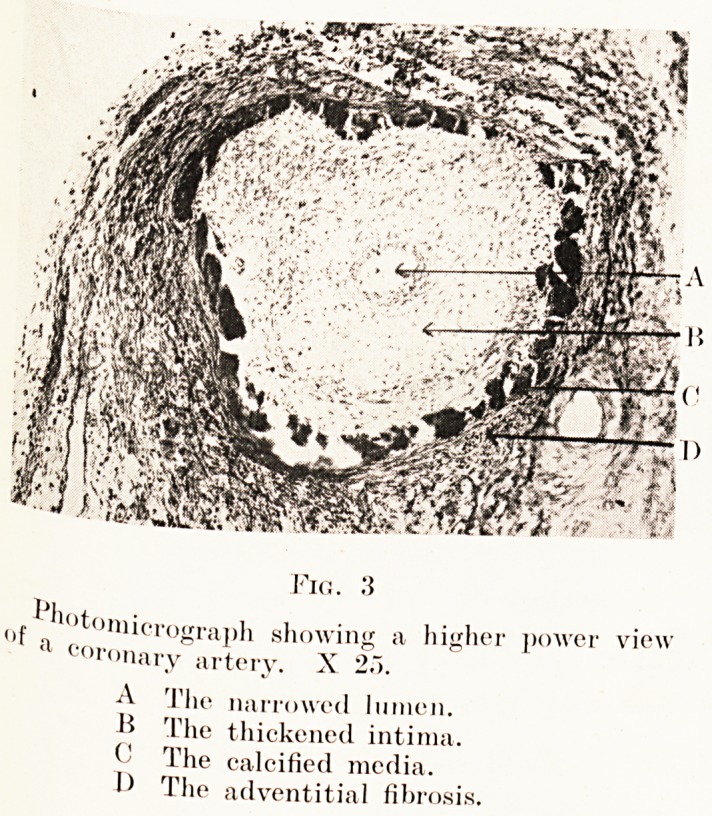


**Fig. 4. f4:**
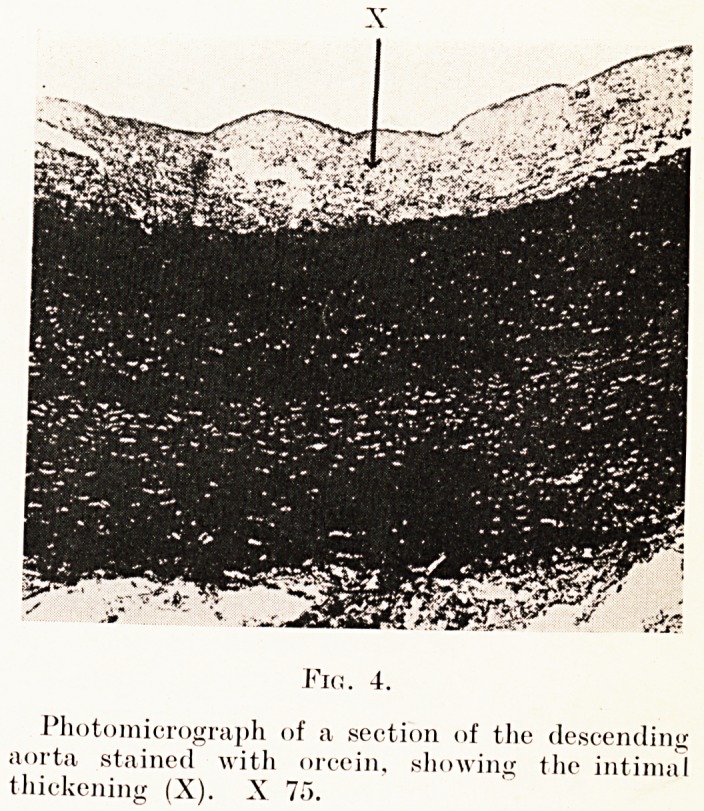


**Fig. 5. f5:**
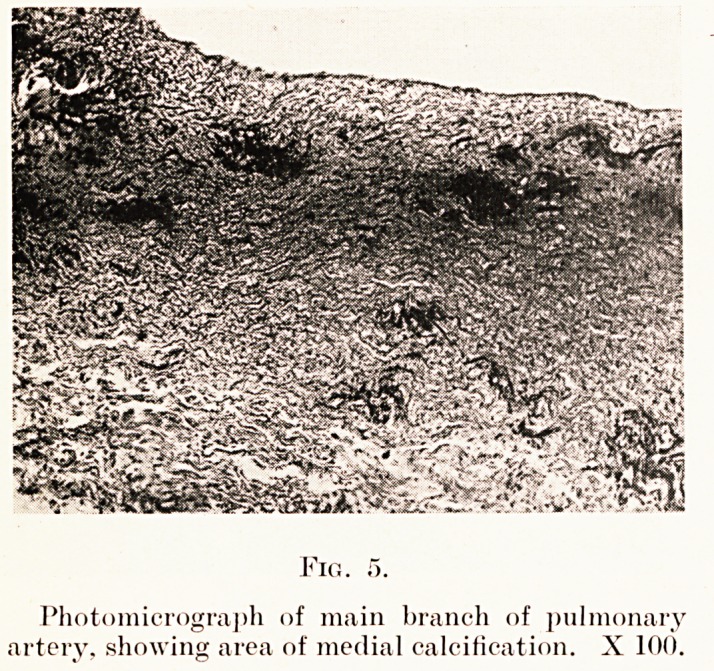


**Fig. 6. f6:**
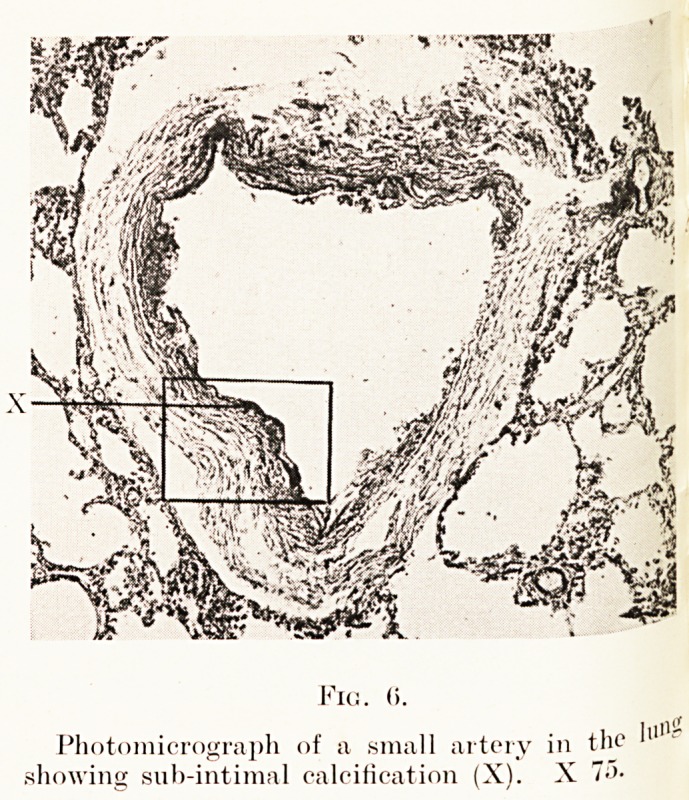


**Fig. 7. f7:**